# Metabolomic Analysis of Aqueous Humor to Predict Glaucoma Progression and Overall Survival After Glaucoma Surgery—The MISO II Study

**DOI:** 10.3390/metabo16020100

**Published:** 2026-01-29

**Authors:** Laurens Detremmerie, Anca Croitor Sava, Uwe Himmelreich, Ingeborg Stalmans, Jan Van Eijgen, João Barbosa-Breda

**Affiliations:** 1 Department of Ophthalmology, University Hospitals UZ Leuven, 3000 Leuven, Belgium; laurens.detremmerie@uzleuven.be (L.D.); ingeborg.stalmans@mac.com (I.S.); jan.1.vaneijgen@uzleuven.be (J.V.E.); 2 Biomedical MRI Unit, Department Imaging and Pathology, KU Leuven, 3000 Leuven, Belgium; anca.croitor@kuleuven.be (A.C.S.); uwe.himmelreich@kuleuven.be (U.H.); 3 Research Group Ophthalmology, Department of Neurosciences, KU Leuven, 3000 Leuven, Belgium; 4 Department of Ophthalmology, ULS São João, 4200-319 Porto, Portugal; 5 RISE-Health, Faculty of Medicine, University of Porto, 4200-319 Porto, Portugal

**Keywords:** aqueous humor, biomarkers, disease progression, glaucoma, metabolomics

## Abstract

Background/Objectives: Although advances in understanding glaucoma have been made, early detection remains challenging due to the asymptomatic nature of the disease. The Metabolomics In Surgical Ophthalmological Patients (MISO) study previously demonstrated that aqueous humor (AH) metabolomics can distinguish glaucoma patients from controls. We aimed to determine if the metabolic profile of AH has predictive power for overall survival and glaucoma progression after surgery. Methods: Glaucoma patients (*n* = 34) were retrospectively analyzed and classified into progression categories based on surgical and medical interventions and assessed for survival. Results: Glutamine and α-ketoglutarate were significantly associated with glaucoma progression, while N-acetylglutamate, lysine, and creatine correlated with mortality. These metabolites are linked to excitotoxicity, mitochondrial dysfunction, and oxidative stress, highlighting their potential role in glaucoma pathophysiology. Conclusions: These results suggest that metabolomic profiling of AH could provide valuable biomarkers for predicting surgical outcomes and overall survival, paving the way for individualized therapeutic approaches. Further studies are required to confirm these findings before they can be integrated into clinical practice.

## 1. Introduction

Glaucoma is the leading cause of irreversible blindness worldwide [1]. The chronic and progressive loss of retinal ganglion cells and their fibers are typically characterized by excavation of the optic disc and associated visual field defects. The only modifiable risk factor of the disease is the intraocular pressure (IOP). Interestingly, a vast number of people exhibit an increased IOP but never develop glaucoma (ocular hypertension—OHT), and vice versa; 30% of all primary open-angle glaucoma (POAG) patients have never had an increased IOP (normal tension glaucoma—NTG). Other risk factors than IOP must therefore be considered, including family history, age, Black race, myopia, and cardiovascular risk factors such as diabetes mellitus [1]. The pathogenesis of POAG is multifactorial, involving vascular dysregulation, mitochondrial dysfunction, oxidative stress, impaired cellular metabolism, and excitotoxicity [2]. Although advances in understanding the disease have been made, early detection remains challenging due to the asymptomatic nature of the disease.

Metabolomics, the study of metabolites in tissues and biofluids, holds promise for identifying potential biomarkers. The MISO study showed that the metabolomic profiling of AH can accurately distinguish glaucoma patients from controls with an AUC of 0.93, serving as a molecular biomarker for glaucomatous neuropathy [3].

In this study, we use metabolites previously extracted from NMR spectra of AH of MISO glaucoma patients to explore their predictive power for overall survival and glaucoma progression after surgery.

Our hypothesis is that AH metabolites that are present in glaucoma patients could potentially reveal underlying processes that drive progression of optic neuropathy, which could direct future research, eventually leading to new and individualized targeted therapies.

## 2. Materials and Methods

### 2.1. Study Participants

A total of 34 glaucoma patients were retrospectively included, of whom 19 were diagnosed with POAG and 15 with NTG (Table 1 and Table 2).

### 2.2. Ethics Statement

The study was approved by the Institutional Review Board of the University Hospitals Leuven (S59677) and adhered to the tenets of the Declaration of Helsinki. All patients provided informed consent prior to study evaluation. The study is registered on clinicaltrials.gov with the ID NCT03098316.

### 2.3. Data Collection

If follow-up of the study participant took place at University Hospitals UZ Leuven, all data was collected from their electronic health record. Of those that were followed up with externally, the relevant data were obtained by sending a questionnaire to the respective ophthalmologist. Of the 54 glaucoma patients included in the initial MISO study, we were able to retrieve follow-up data for 34 patients. Twenty patients were lost to follow-up as they did not return to University Hospitals UZ Leuven, or we were unable to reach their ophthalmologist.

### 2.4. Data Processing and Analysis

Visual Field (VF) Mean Deviation (MD) and MD change per year were extracted. Since some of the patients were followed up externally, considerably less reliable VF data or optical coherence tomography (OCT) data were available for them. Within UZ Leuven, optic nerve evaluation changed from Heidelberg Retinal Tomography^®^ to Heidelberg Spectralis^®^ (Heidelberg Engineering, Heidelberg, Germany) in 2021, which hampered longitudinal OCT assessment of the MISO participants.

**Figure 1:**
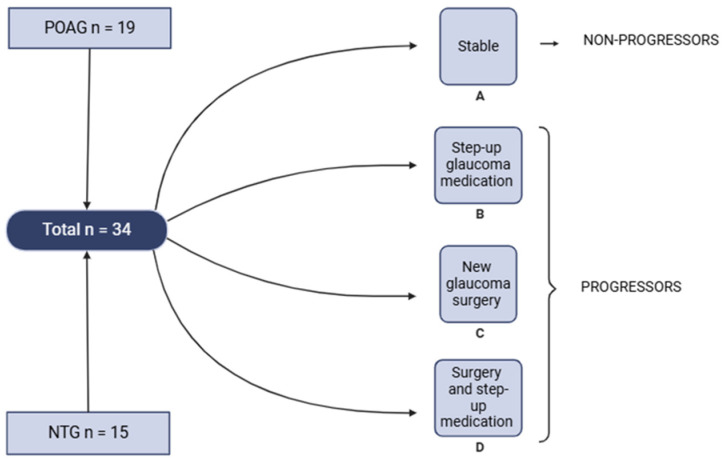
Schematic representation of the included groups.

In this study, three different main analyses were performed. In the first statistical analysis, study participants were labeled as progressors if they needed additional glaucoma surgery and/or increases in glaucoma medication during the follow-up time after the primary glaucoma surgery. If not, patients were labeled non-progressors (Figure 1).

Secondly, study participants who showed progression were further divided into three different categories. When only a step-up in glaucoma medication or only new glaucoma surgery was needed, study subjects were, respectively, assigned to group B (only medication) or group C (only surgery). Group D (medication and surgery) consisted of patients who needed both surgery and a step-up in medication during follow-up. Non-progressors were categorized in group A (stable) (Figure 1).

Thirdly, a binary statistical analysis focused on whether patients had passed away during follow-up.

Study participants were only included in group A (stable) if they had a follow-up time after initial surgery of at least one year. The mean follow-up time for that group was 4.75 years. The mean follow-up time for the patients with any kind of classified progression (groups B, C, and D) was 409 years, and no minimal cut-off for the follow-up period was used. More specifically, the mean follow-up time was 2.73 years for group B (only medication), 4.18 years for group C (only surgery), and 5.54 years for group D (medication and surgery).

Phacoemulsification during follow-up was not seen as surgery for glaucoma progression, nor were intravitreal injection, anterior chamber repair, or suturolysis, as the latter two were part of primary trabeculectomy. Surgery for glaucoma progression included additional trabeculectomy, XEN gel stent^®^ implantation, bleb needling, or bleb needling revision (with lifting of the scleral flap). A step-up in medication was defined as the addition of any IOP-lowering medication class.

The spectral regions of NMR data collected from AH that contributed to the differentiation between glaucoma subjects and healthy controls were collected from the MISO study for each patient (experimental parameters documented in the initial MISO study [3]. To investigate if the spectral regions and their representative metabolites were associated with the failure rate of glaucoma surgery, three statistical analyses were performed using Python (version 3.10.4, Numpy library). Normality was assessed with the Shapiro–Wilk test for every region and a descriptive analysis of the variables in this study was conducted with Python. Outliers were determined using the 3 IQR rule; all analyses were performed with and without outliers. Parametric tests were performed for each region where the data had a normal distribution, using *t*-tests or one-way ANOVA. Non-parametric tests, such as the Mann–Whitney U or Kruskal–Wallis test, were used when the data was non-normally distributed. A correction for age was performed by ANCOVA, with age as a covariate. Continuous variables were reported as means, and categorical variables were documented as proportions. Statistical significance was considered based on two-sided *p*-value < 0.05.

## 3. Results

### 3.1. Population Characteristics

The descriptive characteristics of the included cohort can be read in Table 1.

**Table 1:** Population Characteristics.

	POAG (*n* = 19)	NTG (*n* = 15)	ALL (*n* = 34)
Age (years)	72	70	71
Female *n* (%)	11 (58)	13 (87)	24 (70)
Smoking *n* (%)			
No	12 (63)	8 (53)	20 (59)
Current	2 (11)	3 (20)	5 (15)
Previous	5 (26)	4 (27)	9 (26)
Main systemic diseases			
Arterial hypertension *n* (%)	9 (47)	8 (53)	15 (50)
Hyperlipidemia *n* (%)	6 (32)	5 (33)	11 (33)
Heart surgery *n* (%)	3 (16)	1 (7)	4 12)
Transient ischemic attack *n* (%)	0 (0)	1 (7)	1 (3)
Migraine *n* (%)	1 (5)	1 (7)	2 (6)
Cancer *n* (%)	1 (5)	3 (20)	4 (12)
Psoriasis *n* (%)	0 (0)	1 (7)	1 (3)
Rheumatoid arthritis *n* (%)	1 (5)	1 (7)	2 (6)
Thyroid disease *n* (%)	1 (5)	3 (20)	4 (12)
Ocular characteristics			
Previous surgery *n* (%)			
Trabeculectomy	8 (42)	13 (87)	21 (62)
XEN gel stent^®^	9 (47)	2 (13)	11 (32)
Phacoemulsification	2 (11)	0 (0)	2 (6)
Surgery during trial *n* (%)			
Phacoemulsification	7 (37)	5 (33)	12 (35)
Previous laser *n* (%)			
YAG iridotomy	1 (5)	0 (0)	1 (3)
YAG capsulotomy	1 (5)	2 (13)	3 (9)
Selective laser trabeculoplasty	1 (5)	0 (0)	1 (3)
IOP lowering meds *n* (%)	18 (95)	14 (93)	32 (94)
PGA *n* (%)	16 (84)	13 (87)	29 (85)
BB *n* (%)	3 (16)	0 (0)	3 (9)
CAI *n* (%)	1 (5)	0 (0)	1 (3)
AA *n* (%)	2 (10)	2 (13)	4 (12)
BB_PGA *n* (%)	2 (10)	0 (0)	1 (3)
BB_CAI *n* (%)	12 (63)	13 (87)	25 (74)
BB_AA *n* (%)	0 (0)	0 (0)	0 (0)
AA_CAI *n* (%)	0 (0)	0 (0)	0 (0)
CAI oral *n* (%)	0 (0)	0 (0)	0 (0)

PGA—prostaglandin analog; BB—beta-blocker; CAI—carbonic anhydrase inhibitor; AA—alfa-agonist; BB_PGA—fixed combination of BB and PGA; BB_CAI—fixed combination of BB and CAI; BB_AA—fixed combination of BB and AA; AA_CAI—fixed combination of AA and CAI; CAI oral—oral carbonic anhydrase inhibitor. Variables presented as absolute number (proportion in %).

### 3.2. Systemic Medication Use

The use of systemic medication is summarized in Table 2.

**Table 2:** Systemic Medication Use.

Systemic Medication	POAG (*n* = 19)	NTG (*n* = 15)	ALL (*n* = 34)
Calcium supplements *n* (%)	0 (0)	1 (7)	1 (3)
Magnesium supplements *n* (%)	1 (5)	1 (7)	2 (6)
Vitamin D supplements *n* (%)	0 (0)	2 (13)	0 (0)
Allopurinol *n* (%)	2 (11)	0 (0)	2 (6)
Antihistamine *n* (%)	1 (5)	0 (0)	1 (3)
Steroid *n* (%)	2 (11)	0 (0)	2 (6)
Anticoagulant *n* (%)	2 (11)	0 (0)	2 (6)
Aspirin *n* (%)	1 (5)	1 (7)	2 (6)
Statin *n* (%)	6 (32)	4 (27)	10 (29)
Antihypertensives *n* (%)	9 (47)	8 (53)	17 (50)
BB *n* (%)	4 (21)	4 (27)	8 (24)
CCB *n* (%)	3 (16)	3 (20)	6 (18)
ACEI *n* (%)	3 (16)	3 (20)	6 (18)
ARB *n* (%)	3 (16)	2 (13)	5 (15)
Diuretics *n* (%)	4 (21)	2 (13)	6 (18)
Thyroid hormone *n* (%)	1 (5)	2 (13)	3 (9)
SSRI *n* (%)	0 (0)	2 (13)	2 (6)
Benzodiazepines *n* (%)	2 (11)	2 (13)	4 (12)
Proton pump inhibitors *n* (%)	5 (26)	1 (7)	6 (18)

BB—beta-blocker; CCB—calcium channel blocker; ACEI—angiotensin-converting-enzyme inhibitor; ARB—Angiotensin II receptor blockers; SSRI—Selective serotonin reuptake inhibitors. Variables presented as absolute number (proportion in %).

### 3.3. Metabolic Characteristics

During the follow-up time after the primary glaucoma surgery, 12 patients of the 34 included study participants showed no glaucoma progression (group A), whereas 22 patients did show glaucoma progression according to the proposed ABCD classification (7 in group B, 9 in group C, and 6 in group D). Taken together, out of 34 patients, 19 did not require surgery during follow-up, and 15 did (6 trabeculectomies, 2 XEN gel stents^®^, and 12 needling/revisions).

During the course of the study, one patient in group A died, whereas three died in the progressor groups.

After correcting for age, only region 8 (related to the presence of glutamine and α-ketoglutarate) was significantly higher in progressors (independent of the category), both when performed with and without outliers (*p* = 0.030 and *p* = 0.034) (Table 3 and Table 4). Additionally, after correction for age, the values for regions 3 (N-acetylglutamate and lysine), 5 (glutamine and glutamate), and 8 (glutamine and α-ketoglutarate) were significantly higher and associated with death, both with and without outliers (respectively, *p* = 0.048, 0.038 and 0.019; *p* = 0.012, 0.038, and 0.019). Region 10 (lysine, creatine, phosphocreatine, creatinine, and α-ketoglutarate) was also significantly higher in progressors and associated with death after age correction in the full dataset with outliers (*p* = 0.023) but lost significance after outlier removal (*p* = 0.085). There were no significant differences in spectral regions between the four distinct categories (group A, B, C, and D, ANOVA and ANCOVA).

## 4. Discussion

Previously, the MISO study demonstrated that NMR spectroscopy of AH can effectively differentiate glaucoma patients from healthy controls [3]. This diagnostic capability using biofluids in glaucoma has been validated in several other metabolomic studies [4,5,6,7,8,9]. In the current analysis, we have shown that these metabolites can not only serve as diagnostic biomarkers for disease detection but also as predictive biomarkers for future disease progression and overall survival.

So far, one meta-analysis has been conducted focusing on the metabolomics of POAG patients in human AH and plasma [10]. From all 18 included studies, six significantly enriched pathways were identified in the AH. The most significantly enriched pathway was aminoacyl-tRNA biosynthesis. D-Glutamine and D-glutamate metabolism was the second most significantly enriched pathway. Others included galactose metabolism, arginine biosynthesis, glycine metabolism, and arginine metabolism. Furthermore, the metabolites lysine, creatine, glycine, and alanine were significantly higher in glaucoma patients than in the control subjects.

The analysis identified four significantly enriched pathways in the plasma of patients with POAG, including arginine, proline, glyoxylate, and dicarboxylate metabolism. Significant changes in plasma methionine, arginine, tyrosine, nicotinamide, and hydroxyproline were reported.

In this meta-analysis, alanine in AH and methionine in plasma were identified as the most reliable biomarkers for POAG presence.

Recently, metabolic profiling of aqueous humor from POAG patients identified metabolites with anti-inflammatory and neuroprotective potential in mice [11]. Agmatine and thiamine were significantly decreased in POAG patients. Topical delivery of agmatine and thiamine significantly reduced the inflammatory response and protected retinal ganglion cell function in the mouse retina.

### 4.1. Glutamate—A (Toxic) Metabolic Hub in the Excitatory Synapse

Our data shows that glutamine and α-ketoglutarate, both connected in the metabolic hub of glutamate, are associated with a higher likelihood of progression and lower overall survival. Glutamate is the main excitatory neurotransmitter and plays a crucial role in visual signal transduction in the retina [12]. Multiple studies have examined its neuroexcitotoxic effects, where activation of N-methyl-D-aspartic acid receptors (NMDARs) causes excessive calcium influx into cells, ultimately resulting in the death of retinal ganglion cells (RGCs) [4,13,14,15].

**Figure 2:**
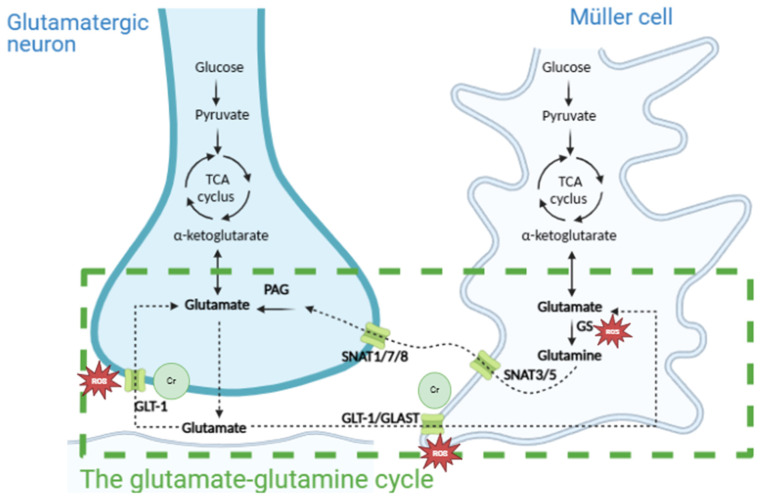
**Visual representation of the glutamate–glutamine cycle.** Full arrows are enzymatic reactions, dotted arrows are referring to transport without molecular changes. The primary substrate for the synthesis of glutamate is glucose. Its synthesis is connected to neuronal metabolism through the tricarboxylic acid cycle (TCA). Glutamate is mainly cleared from the synapse through Müller cell uptake via the GLT-1 and GLAST transporters, while a smaller portion is removed by neuronal uptake, primarily driven by GLT-1 activity. Within Müller cells, glutamate is converted to glutamine by the enzyme glutamine synthetase (GS). Glutamine can then be released from Müller cells through SNAT3/5 transporters and is subsequently taken up by neurons, mainly via SNAT1/7/8. Once inside neurons, glutamine is converted back to glutamate by phosphate-activated glutaminase (PAG), completing the cycle. Glutamate is also oxidatively metabolized in the TCA cycle out of α-ketoglutarate in both neurons and Müller cells [12]. Glutamate transporter proteins and glutamine synthase are modified by ROS, connecting the neurotoxicity of glutamate with the lysine increase caused by mitochondrial dysfunction described in Figure 3 [27]. Creatine (Cr) is essential for glutamate clearance during excitatory synaptic transmission in the brain and provides both direct and indirect antioxidant benefits. Therefore, the increase in creatine could be explained as a potential counterreaction for neuroprotection of RGCs against mitochondrial stress and excitotoxicity [28].

Glutamate and glutamine are extensively recycled between neurons and Müller cells in a process known as the glutamate–glutamine cycle (Figure 2) [16]. This process maintains an adequate glutamate level in the synaptic cleft. It is estimated that around 80% of all glutamate that is taken up is turned into glutamine [17]. Higher concentrations of glutamine and α-ketoglutarate found in the AH of glaucoma patients may be the result of an interruption in this glutamate–glutamine cycle.

The advances in knowledge regarding glutamate metabolism have been discussed in other neurodegenerative diseases [12]. In Alzheimer’s disease (AD), for example, several studies have reported reduced expression of GLT-1 in AD brain tissue, a key transporter in the glutamate–glutamine cycle [18,19]. Amyloid-β (Aβ) seems to directly impair astrocyte glutamate uptake and reduce surface expression of GLT-1. In Huntington’s disease (HD), decreased expression of the glutamine transporter SNAT3 was documented in mouse models, which could explain the reported accumulation of cerebral glutamine in HD [20]. Studies regarding changes in expression or activity of specific glutamine/glutamate and α-ketoglutarate transporters in glaucoma progression have not yet been published.

Interestingly, in mouse models, the susceptibility to NMDA excitotoxicity depended on the type of RGCs involved [21]. αRGCs were the most resistant type of RGCs to NMDA excitotoxicity, while J-RGCs were the most sensitive cells to NMDA excitotoxicity. Not only glutamate and its precursors but also the target RGCs and their different cell types might provide valuable insights into understanding the pathophysiology behind excitotoxicity.

The impact on glaucoma progression and overall survival cannot be attributed to neuroexcitotoxicity alone. A systematic review of 71 included studies focused on the multiple pathogenic mechanisms and toxic effects of glutamate to neurons in the brain in vitro and in vivo [22]. These studies showed glutamate exposure reduced neuronal viability by inducing neuronal degradation and cell apoptosis, and impaired cellular oxidant defense by decreasing activity of superoxide dismutase and catalase [23,24,25]. Furthermore, activation of proinflammatory markers such as nuclear factor kappa-light-chain-enhancer (NF-kB) and cyclooxygenase-2 (COX-2) triggering neuroinflammation has been noticed, leading to neuronal death [26].

### 4.2. Lysine—A Reflector of Mitochondrial Hemostasis

As we discussed in Figure 1, the glutamate–glutamine cycle is closely linked to cellular energy metabolism. Our findings that amino acid lysine, included in regions 3 and 10 in the NMR spectroscopy in the MISO study, is associated with death, even after correcting for age, might point towards an already altered energy metabolism or oxidative state in those patients.

**Figure 3:**
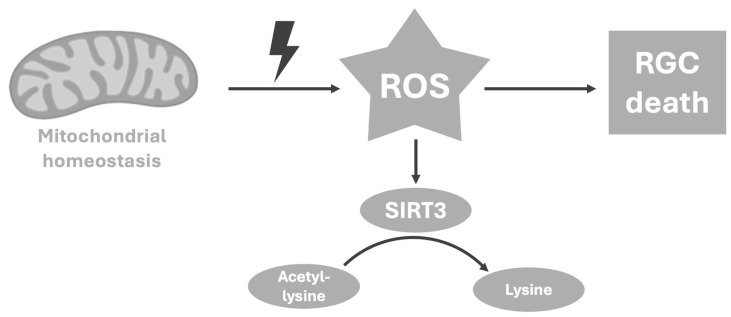
**Disruption of mitochondrial homeostasis leads to ganglion cell death.** A reduction in oxygen supply inhibits oxidative phosphorylation in the mitochondria and disturbs mitochondrial hemostasis, resulting in the generation of reactive oxygen species (ROS), causing oxidative stress and ultimately leading to cell damage and retinal ganglion cell (RGC) death. SIRT3 is a main deacetylase catalyzing the conversion of acetyl-lysine to lysine. SIRT3 levels may increase in response to increased ROS synthesis, which leads to an increase in lysine formation [2,27].

Lysine is an essential amino acid with multiple essential roles in protein synthesis, immune function, collagen formation, and hormone production [29]. It is catabolized in the mitochondria and reflects mitochondrial hemostasis, another pathway in the etiology of POAG that seems to be affected [2]. A reduction in the oxygen supply inhibits oxidative phosphorylation in mitochondria and disturbs mitochondrial hemostasis, resulting in the generation of reactive oxygen species (ROS), causing oxidative stress and ultimately leading to cell damage and RGC death (Figure 3). Oxidative stress is a common symptom of mitochondrial dysfunction and is strongly implicated in the pathogenesis of other neurodegenerative diseases [30]. In AD, for example, mitochondrial ROS increase Aβ levels in cells and mouse models, which in turn causes mitochondrial dysfunction. Additionally, AD patients tend to have a higher number of somatic mutations in the mitochondrial DNA compared to control subjects [31]. In POAG, evidence for lower complex I activity and mitochondrial DNA mutations in complex I has been reported [32,33]. One study investigated how lysine acetylation status can directly alter mitochondrial function [34], which could offer an explanation for the link between mitochondrial dysfunction and the increase in lysine in the AH of glaucoma patients.

Sirtuins (SIRTs) are deacetylases that are involved in the regulation of many cellular activities [27]. SIRT3 is the principal NAD^+^-dependent deacetylase in the mitochondria, catalyzing the conversion acetyl-lysine to lysine. SIRT3 levels may increase in response to increased ROS synthesis, which leads to an increase in lysine formation.

Lastly, glutamate transporter proteins and glutamine synthase, which converts retinal glutamate into a nontoxic form, are modified by ROS in vivo, connecting the neurotoxicity of glutamate, described above, with the lysine increase caused by mitochondrial dysfunction (Figure 2) [28,35]. These findings support that mitochondrial dysfunction, correlated with an increase in lysine in AH, could be a main characteristic in the pathogenesis of glaucoma and contributes to (cell) death.

### 4.3. Creatine—A Possible Neuroprotector as a Counterreaction Against Oxidative Stress

Increased creatine in the AH of glaucoma patients, including in region 10 in the NMR spectroscopy in the MISO study, is associated with death after correction for age (only in the full dataset).

Creatine is produced in the kidneys and liver and is used in the phosphocreatine system to generate ATP. A total of 95% of creatine is stored in the muscles, providing energy by ATP, including the ciliary body [36]. Researchers previously speculated that elevated creatine levels may lead to increased AH production and increased IOP [10].

On the other hand, several studies reported on the neuroprotective effect of creatine. In vitro studies showed pretreatment with creatine 5 mM was able to cause a significant reduction in ROS levels on mixed retinal cultures subject to sodium azide-induced metabolic stress and prevent neuronal loss in stress-derived mitochondrial dysfunction [37]. Other in vivo studies showed creatine prevents intracellular calcium and ROS accumulation [38].

Interestingly, creatine is essential for glutamate clearance during excitatory synaptic transmission in the brain and provides both direct and indirect antioxidant benefits (Figure 1) [28]. Therefore, the increase in creatine could be explained as a potential counterreaction for neuroprotection of RGCs against mitochondrial stress and excitotoxicity, and raises the question of the role of creatine supplementation in glaucoma patients.

In other neurodegenerative diseases such as amyotrophic lateral sclerosis (ALS), creatine supplementation in a mouse model reduced neuronal loss in the motor cortex and substantia nigra, and reduced ROS damage has been reported [39].

However, the efficacy of long-term, high-dose creatine supplementation in patients with neurodegenerative diseases still remains unclear [40]. Moreover, a case study described a case of central retinal vein occlusion associated with creatine supplementation in a 25-year-old man, probably due to dehydration [41].

### 4.4. Methionine—A Circulating Precursor Linked to Metabolic and Vascular Disease

Metabolite changes in glaucoma patients are not only found in AH but are also found to be increased in the corpus geniculatum laterale in the brain and in plasma of patients with POAG [10,13,41]. Remarkably, the plasma level of methionine, a precursor of creatine, is significantly higher in patients with POAG [10]. We have shown that an increase in creatine in AH, including in region 10 in the NMR spectroscopy in the MISO study, is associated with death after correction for age. The pathophysiological process of its possible systemic impact is unknown, but elevated serum methionine levels are correlated with coronary, cerebrovascular, and arterial occlusive diseases [42]. Moreover, restricting methionine intake has been shown to increase lifespan in various species and positively impact metabolic health and inflammatory responses [43].

Given these metabolite changes in systemic circulation and the correlation of these metabolites with death in the AH of glaucoma patients, metabolomics can be an interesting path for new research concerning the overall survival of patients with glaucoma.

Current limitations of our study include a small sample size, which could be reflected by the drop in significance after removal of outliers. Secondly, we were not able to use structural (OCT) or functional (VF) optic nerve evaluations as markers for disease progression given the relatively short follow-up period and the heterogeneity of devices within UZ Leuven and between UZ Leuven and private practices. Therefore, our glaucoma progression definition pragmatically relied on therapy changes as deemed necessary by a glaucoma expert, based on a variety of inputs. As mentioned in the Methods, we included study participants in group A (stable) if progression was absent in their follow-up period (median was 6.04 years), which cannot fully exclude future progression after that window. Our progression criteria are based on the decision-making of a glaucoma specialist, who had access to multimodal information (not solely based on IOP). There is a follow-up bias towards progressive patients, as those who were followed up externally, were referred back to UZ Leuven when progression was detected.

## 5. Conclusions

In this study, we have shown that the metabolomic profile of AH is distinct and predictive of future glaucoma progression and death during follow-up.

The presence of glutamine and α-ketoglutarate in AH was significantly higher in glaucoma patients who needed additional glaucoma surgery or a step-up in glaucoma medication during the follow-up time after the primary glaucoma surgery (independent of progression category). This might be due to its neuroexcitotoxic effects inducing neuronal degradation and cell apoptosis, resulting in the death of RGCs.

Additionally, after correcting for age, N-acetylglutamate, lysine, glutamine, and α-ketoglutarate were significantly higher and associated with death. This may be linked with a disturbance in mitochondrial hemostasis and the generation of ROS, causing oxidative stress and cell death.

Lastly, lysine, creatine, phosphocreatine, creatinine, and α-ketoglutarate were significantly higher and associated with death after age correction in the full dataset with outliers but lost significance after outlier removal.

These metabolite alterations point to new targets for individualized and metabolomics-driven therapies or nutrient supplementation in glaucoma patients. In the future, anterior chamber tap and analysis of aqueous humor may be considered as a potential screening approach or part of the work-up at initial diagnosis. Glaucoma is more common in elderly people, most of whom need to undergo cataract surgery. When this occurs, instead of discarding the aqueous humor, it can potentially be used for metabolomics analysis.

In addition, the potential of AH NMR spectroscopy to predict surgical outcomes has been demonstrated, similar to other potential diagnostic applications of NMR spectroscopy in body fluids [44,45,46]. Nonetheless, validation in larger patient cohorts must be conducted.

## Figures and Tables

**Table 3:** Main metabolites that contribute to these regions. *p*-value is reported. Model corrected for age; ANCOVA; with outliers; bold if significant.

Region	Chemical Shift Region	Main Metabolites	Progression; *p*-Value	Progression Category; *p*-Value	Death; *p*-Value
1	1.48–1.54	Alanine	0.151	0.243	0.064
2	1.55–1.65	Lysine, leucine	0.248	0.472	0.632
3	1.88–1.92	N-acetylglutamateLysine	0.242	0.309	**0.048**
4	1.93–1.95	Acetate	0.317	0.806	0.592
5	2.1–2.2	Glutamine and glutamate	0.081	0.212	**0.038**
6	2.22–2.25	Valine, β-hydroxybutyrate	0.855	0.373	0.534
7	2.38–2.42	Glutamate, succinate, β-hydroxybutyrate	0.337	0.171	0.628
8	2.44–2.52	Glutamine, α-ketoglutarate	**0.030**	0.176	**0.019**
9	2.5–2.8	Citrate	0.635	0.3945	0.954
10	3.05–3.1	Lysine, creatine, phosphocreatine, creatinine, α-ketoglutarate	0.176	0.605	**0.023**
11	3.24–3.32	Glucose, taurine, betaine	0.250	0.517	0.072
12	3.4–3.98	Glucose and Hα of amino acids	0.578	0.890	0.263
13	4.0–4.08	β-hydroxybutyrate	0.260	0.692	0.258
14	4.1–4.2	Lactate	0.362	0.843	0.674

**Table 4:** Main metabolites that contribute to these regions. *p*-value is reported. Model corrected for age; ANCOVA; without outliers; bold if significant.

Region	Chemical Shift Region	Main Metabolites	Progression; *p*-Value	Progression Category; *p*-Value	Death; *p*-Value
1	1.48–1.54	Alanine	0.265	0.431	0.454
2	1.55–1.65	Lysine, leucine	0.456	0.195	0.555
3	1.88–1.92	N-acetylglutamateLysine	0.337	0.561	**0.012**
4	1.93–1.95	Acetate	0.501	0.831	0.226
5	2.1–2.2	Glutamine and glutamate	0.081	0.212	**0.038**
6	2.22–2.25	Valine, β-hydroxybutyrate	0.985	0.578	0.883
7	2.38–2.42	Glutamate, succinate, β-hydroxybutyrate	0.608	0.466	0.978
8	2.44–2.52	Glutamine, α-ketoglutarate	**0.034**	0.176	**0.019**
9	2.5–2.8	Citrate	0.051	0.204	0.557
10	3.05–3.1	Lysine, creatine, phosphocreatine, creatinine, α-ketoglutarate	0.358	0.828	0.085
11	3.24–3.32	Glucose, taurine, betaine	0.250	0.517	0.072
12	3.4–3.98	Glucose and Hα of amino acids	0.578	0.890	0.263
13	4.0–4.08	β-hydroxybutyrate	0.344	0.604	0.186
14	4.1–4.2	Lactate	0.689	0.551	0.924

## Data Availability

The original contributions presented in this study are included in the initial MISO study [3]. Further inquiries can be directed to the corresponding author.

## Abbreviations

The following abbreviations are used in this manuscript:

MISO	The Metabolomics In Surgical Ophthalmological Patients Study
AH	Aqueous humor
IOP	Intraocular pressure
OHT	Ocular hypertension
POAG	Primary open-angle glaucoma
NTG	Normal tension glaucoma
VF	Visual field
MD	Mean deviation
NMDARs	N-methyl-D-aspartic acid receptors
RGCs	Retinal ganglion cells
AD	Alzheimer’s disease
Aβ	Amyloid-β
HD	Huntington’s disease
NF-kB	Nuclear factor kappa-light-chain-enhancer
COX-2	Cucmppcygenase-2
TCA	Tricarboxylic acid cycle
GS	Glutamine synthetase
PAG	Phosphate-activated glutaminase
ROS	Reactive oxygen species
SIRT	Sirtuin
ALS	Amyotrophic lateral sclerosis
